# Comparative clinical outcomes of different therapies for traumatic meniscal tears in adults: A protocol for systematic review and network meta-analysis

**DOI:** 10.1097/MD.0000000000028557

**Published:** 2022-01-14

**Authors:** Jun-Hu Hou, Yan-Long Gong, Ping Ma, Xin Chen, Wan-Tao Dong, Jian-Jun Liu, Bao-Jian Liu, Chun-Mu Zhang

**Affiliations:** Department of Sports Medicine, Affiliated Hospital of Gansu University of Traditional Chinese Medicine, Lanzhou, China.

**Keywords:** different therapies, network meta-analysis, protocol, systematic review, traumatic meniscal tears

## Abstract

**Background:**

Meniscus tears are usually classified as degenerative or traumatic tears according to their pathogenesis. At present, traumatic meniscal tears are generally believed to have high healing potential. In recent years, multiple treatments have been described for traumatic meniscal tears, such as the inside-out technique, outside-in technique, all-inside technique, biological augmentation of meniscal repair, meniscectomy, and non-surgical treatment. However, the functional recovery of the knee joint and healing of the meniscus after treatment are quite different from the results reported in the literature, which requires more reliable evidence-based medical findings. This study will evaluate evidence from multiple types of research comparing different therapies for traumatic meniscal tears in adults.

**Methods:**

We will search the EMBASE, Cochrane Library (the Cochrane Database of Systematic Reviews, the Cochrane Central Register of Controlled Trials [CENTRAL], Cochrane Methodology Register), PubMed, Web of Science (Science and Social Science Citation Index), China Knowledge Network, CBM, Wanfang data, and VIP electronic databases from their inception to August 10, 2021, with no language restrictions. We will also manually search Baidu and Google Scholar to identify randomized controlled studies, non-randomized controlled studies, and cohort studies on the treatment of traumatic meniscal tears. Two researchers will independently screen the literature, extract the data, and evaluate the quality of the studies. Software programs, including Microsoft Access, Excel, Stata (Version 15), WinBUGS (Version 1.4.3), and ADDIS (Version 1.16.8), were used to analyze and manipulate the data.

**Results:**

In this study, the main outcomes were physical function and healing rate, based on the Western Ontario and McMaster Universities Osteoarthritis Index, Lysholm Knee Scoring Scale, Knee Injury and Osteoarthritis Outcome Score, Functional Recovery Scale, and clinical healing rate. The secondary indexes included total cost, cost-effectiveness ratio, incremental cost-effectiveness ratio, Tegner activity scale score, visual analogue scale, numerical rating scale, and meniscal tear complications.

**Conclusions:**

This systematic review will provide reliable evidence-based findings for the clinical application of different therapies for traumatic meniscal tears in adults.

## 1 Introduction

Meniscus tears are one of the most common musculoskeletal injuries, which often cause pain and dysfunction. These tears can be divided according to their pathogenesis into degenerative tears and traumatic tears. The established concept is that the meniscus tissue should be preserved as much as possible to prevent knee degeneration.^[1]^ However, meniscal tears cannot always be repaired. While the healing rate is low in degenerative tears, traumatic tears show a higher healing rate after repair owing to the better condition of the meniscus tissue.

Current research shows that traumatic meniscal tears occur in 0.06% to 0.07% of adults, with meniscal tears comprising nearly 50% of knee injuries. Biomechanical studies emphasize the important load-bearing and shock-absorbing functions of the meniscus in the human knee. Thus, meniscus repair plays an active role in the restoration of knee function by maintaining tissue integrity.^[2,3]^

In recent years, multiple treatments have been described for traumatic meniscal tears, specifically the inside-out technique, outside-in technique, all-inside technique, biological augmentation of meniscal repair, meniscectomy, and non-surgical treatment.^[4]^ However, the functional recovery of the knee joint and healing of the meniscus after treatment are quite different from those reported in the literature.^[5–7]^ Thus, more reliable evidence-based medical findings is required for reference. This study aims to evaluate all evidence from multiple types of research comparing different therapies for traumatic meniscal tears in adults. While there are many pairwise comparative studies of treatments for traumatic meniscal tears, there are few high-quality network meta-analysis studies.^[8–11]^ The ranking of the efficacy and cost-effectiveness of multiple treatments is also unclear. Therefore, this systematic review and network meta-analysis will provide important information for clinical decision-making and as the main source of evidence for the development of treatment guidelines.

## 2 Methods

### 2.1 Protocol registration

This protocol was drafted based on the Preferred Reporting Items for Systematic Reviews and Meta-analysis Protocols (PRISMA-P).^[12,13]^ Additionally, this study was registered in PROSPERO on August 8, 2021 (registration number: CRD42021272353).

### 2.2 Ethics

Since this study protocol does not contain personal information or perform patient recruitment, it does not involve ethical issues.

### 2.3 Inclusion criteria for study selection

#### 2.3.1 Research type

We will include all studies comparing the different therapies for traumatic meniscal tears in adults, including randomized controlled trials (RCTs), non-RCTs, and observational studies with a comparator arm. There are no language restrictions. Other studies, such as animal studies, case reports, and reviews, will be excluded.

#### 2.3.2 Participants

We will include adults aged ≥18 years who were diagnosed with traumatic meniscal tears, regardless of their nationality, sex, and tear location.

#### 2.3.3 Interventions

The main interventions are classified into the following 6 categories: inside-out technique,^[14]^ outside-in technique,^[14]^ all-inside technique,^[15]^ biological augmentation of meniscal repair, meniscectomy (arthroscopic partial or total meniscectomy), and non-surgical treatment.

#### 2.3.4 Outcome measures

The main outcomes are physical function and healing rate, based on the Western Ontario and McMaster Universities Osteoarthritis Index,^[16]^ Lysholm Knee Scoring Scale,^[17]^ Knee Injury and Osteoarthritis Outcome Score,^[18]^ Functional Recovery Scale, and clinical healing rate. The secondary indexes include total cost, cost-effectiveness ratio, incremental cost-effectiveness ratio, Tegner activity scale score, visual analogue scale, numerical rating scale, and complications.

### 2.4 Exclusion criteria

(1) Patients with moderate to severe osteoarthritis.
(2) Letters, conference papers, descriptive research, and animal research.
(3) Duplicate results, repeated publications, or unavailability of full text.
(4) Critical data not available.

### 2.5 Search strategy

We will search the following electronic databases: EMBASE, the Cochrane Library (the Cochrane Database of Systematic Reviews, Cochrane Central Register of Controlled Trials (CENTRAL), and Cochrane Methodology Register), PubMed, Web of Science (Science and Social Science Citation Index), China Knowledge Network, CBM, Wanfang data, and VIP. The search dates are from inception to August 10, 2021. There are no language restrictions. We will also manually search Baidu and Google Scholar to identify RCTs, non-RCTs, and cohort studies on the treatment of traumatic meniscal tears. We will not establish a limitation on publication status. The search strategy including MeSH terms and keywords is as follows:(“Tibial Meniscus Injuries” [Mesh] OR “Menisci, Tibial” [Mesh] OR “Meniscus” [Mesh] OR “Meniscus” [All Fields] OR “menisci” [All Fields] OR “meniscus injury” [All Fields] OR “Meniscal Tear” [All Fields] OR “meniscus tear injury” [All Fields] OR “ears of menisci” [All Fields] OR “meniscal tears” [All Fields] OR “meniscus tear” [All Fields] OR “Torn Meniscus” [All Fields]) AND (“Combined Modality Therapy” [Mesh] OR “Exercise Therapy” [Mesh] OR “Physical Therapy Modalities” [Mesh] OR “Musculoskeletal Manipulations” [Mesh] OR “Drug Therapy” [Mesh] OR “Physical and Rehabilitation Medicine” [Mesh] OR “Conservative Treatment” [All Fields] OR “Therapy” [All Fields] OR “Inside-Out Technique” [All Fields] OR “Outside-In Technique” [All Fields] OR “All-Inside Technique” [All Fields] OR “biological augmentation” [All Fields] OR “meniscectomy” [All Fields] OR “sham operation” [All Fields] OR “placebo” [All Fields]) AND ((“Randomized Controlled Trials as Topic” [Mesh] OR “Randomized Controlled Trial” [Publication Type] OR “Controlled Clinical Trial” [Publication Type]” OR “Controlled Clinical Trials as Topic” (Mesh) OR (“Non-Randomized Controlled Trials as Topic” [Mesh] OR “Non-Randomized Controlled Trial” [Publication Type]) OR (“Cohort Studies” [Mesh] OR “Cohort Study” [Publication Type])).

### 2.6 Data screening and extraction

**Figure 1:**
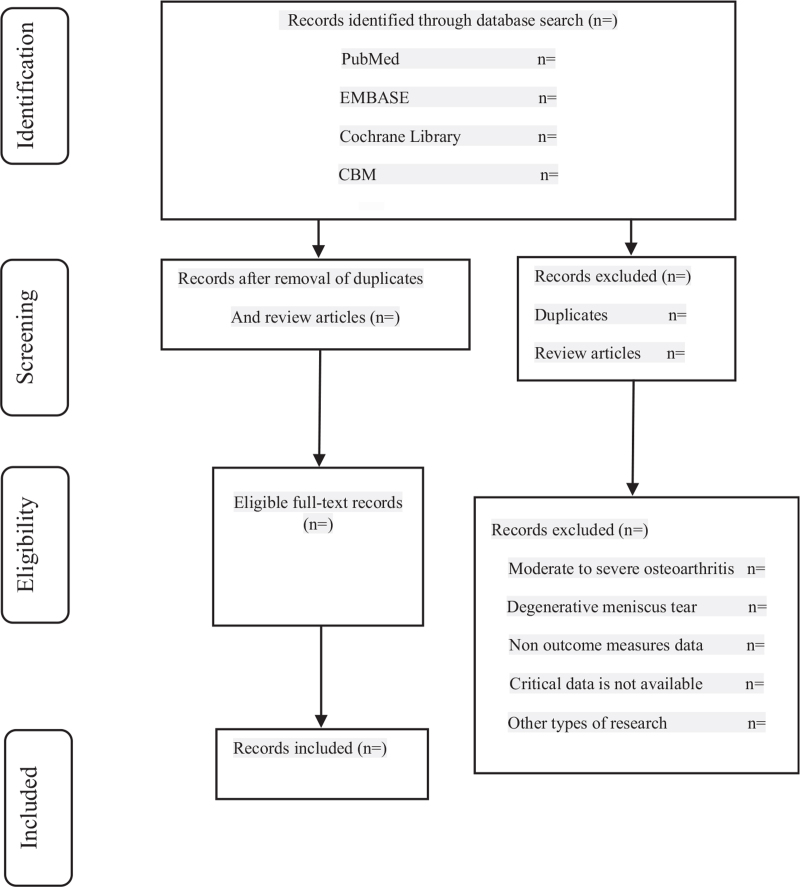
Study search and selection.

According to the PRISMA flow chart,^[19]^ we will refer to the Cochrane Collaborative System Evaluator's Manual 5.0 to evaluate the research quality and filter the literature based on the exclusion and inclusion criteria. Two authors will independently search for pertinent literature by reading titles, abstracts, and full texts, thus selecting manuscripts and extracting data from the included studies. In cases of disagreement, another reviewer will decide. The documents retrieval process for Chinese and foreign language databases is shown in Fig. 1.

### 2.7 Literature quality evaluation

We will use the Cochrane risk-of-bias tool to assess the quality of the literature,^[20]^ which includes the following: randomization process, allocation concealment, blind method, missing outcome data, measurement of the outcome, selection of the reported result, and other biases.

### 2.8 Statistical analysis

#### 2.8.1 Data analysis and processing

Two reviewers will independently evaluate the quality of each trial using the Cochrane Risk of Bias assessment tool. We will use Stata SE V.14.2 (Stata, College Station, TX) and Revman V.5 2008 (The Cochrane Collaboration, Copenhagen, Denmark) to perform the statistical analyses. The primary network meta-analysis will use a Bayesian framework. Odds ratios and 95% confidence intervals will be used to analyze dichotomous data, while continuous variables will be analyzed according to the mean differences and 95% confidence intervals.

Statistical significance is set at *P* < .05. A plot of the surface under the cumulative ranking curve (SUCRA) will be generated using STATA to indicate the probability of each intervention being ranked best.^[21]^ SUCRA values of 100% and 0% indicate that a treatment is certain to be the best and worst, respectively. We will assess the risk of publication bias using funnel plots and Egger tests.^[22,23]^

#### 2.8.2 Missing data

We will contact the authors to obtain the missing data. If the data cannot be obtained, we will perform descriptive analysis instead of network meta-analysis.

#### 2.8.3 Subgroup analysis

If conditions permit, we will perform further excess subgroup analyses based on the results of heterogeneity and inconsistency (such as tear type, treatment time window, and age).

#### 2.8.4 Sensitivity analysis

To ensure the stability of the merged results, we will perform a sensitivity analysis of each outcome index by excluding studies with a high risk of bias.

#### 2.8.5 Evidence quality

We will use the grades of recommendation assessment development and evaluation (GRADE) guidelines to assess the quality of evidence for all outcomes. This includes the following 5 factors: inconsistency, risk of bias, indirectness, inaccuracy, publication bias, and the quality will be graded as very low, low, moderate, or high.

## 3 Discussion

Among the existing treatments for traumatic meniscal tears, the most widely used are the inside-out technique, outside-in technique, all-inside technique, biological augmentation of meniscal repair, meniscectomy, and non-surgical treatment. Current data suggest that traumatic meniscal tears have a high healing potential.^[24]^ This is the first network meta-analysis to compare the efficacy, safety, and cost-effectiveness of different therapies for traumatic meniscal tears in adults. We will generate a treatment ranking based on the observed outcomes. A descriptive analysis will be used if the data cannot be merged.

## Author contributions

**Conceptualization:** Junhu Hou.

**Data curation:** Junhu Hou.

**Formal analysis:** Yanlong Gong.

**Investigation:** Ping Ma, Xin Chen.

**Methodology:** Junhu Hou, Chunmu Zhang.

**Resources:** Wantao Dong, Jianjun Liu, Baojian Liu.

**Software:** Yanlong Gong.

**Supervision:** Junhu Hou, Chunmu Zhang.

**Validation:** Xin Chen, Jianjun Liu.

**Visualization:** Ping Ma.

**Writing – original draft:** Junhu Hou, Yanlong Gong.

**Writing – review & editing:** Junhu Hou, Chunmu Zhang.
